# Early thrombocytopenia is associated with an increased risk of mortality in patients with traumatic brain injury treated in the intensive care unit: a Finnish Intensive Care Consortium study

**DOI:** 10.1007/s00701-022-05277-9

**Published:** 2022-07-15

**Authors:** Kadri Lillemäe, Teemu Luostarinen, Matti Reinikainen, Stepani Bendel, Ruut Laitio, Sanna Hoppu, Tero Ala-Kokko, Tomi Niemi, Markus B. Skrifvars, Rahul Raj

**Affiliations:** 1 Department of Anesthesiology, Intensive Care, Emergency and Pain Medicine, Helsinki University Hospital and University of Helsinki, Helsinki, Finland; 2 HUS, Töölö Hospital, Topeliuksenkatu 5, P.B. 266, 00029 Helsinki, Finland; 3 Anesthesiology and Intensive Care, Hyvinkää Hospital, Helsinki University Hospital and University of Helsinki, Helsinki, Finland; 4 Department of Anesthesiology and Intensive Care, Kuopio University Hospital and University of Eastern Finland, Kuopio, Finland; 5 Department of Perioperative Services, Intensive Care and Pain Management, Turku University Hospital and University of Turku, Turku, Finland; 6 Department of Intensive Care and Emergency Medicine Services, Tampere University Hospital and Tampere University, Tampere, Finland; 7 Department of Intensive Care and MRC Oulu, Research Group of Surgery, Anesthesiology and Intensive Care Medicine, Oulu University Hospital, University of Oulu, Oulu, Finland; 8 Department of Emergency Care and Services, University of Helsinki and Helsinki University Hospital, Helsinki, Finland; 9 Department of Neurosurgery, Helsinki University Hospital and University of Helsinki, Helsinki, Finland

**Keywords:** Traumatic brain injury, Low platelet count, Thrombocytopenia, Platelet transfusion, Long-term outcome, One-year mortality

## Abstract

**Background:**

Coagulopathy after traumatic brain injury (TBI) is associated with poor prognosis.

**Purpose:**

To assess the prevalence and association with outcomes of early thrombocytopenia in patients with TBI treated in the intensive care unit (ICU).

**Methods:**

This is a retrospective multicenter study of adult TBI patients admitted to ICUs during 2003–2019. Thrombocytopenia was defined as a platelet count < 100 × 10^9^/L during the first day. The association between thrombocytopenia and hospital and 12-month mortality was tested using multivariable logistic regression, adjusting for markers of injury severity.

**Results:**

Of 4419 patients, 530 (12%) had early thrombocytopenia. In patients with thrombocytopenia, hospital and 12-month mortality were 26% and 48%, respectively; in patients with a platelet count > 100 × 10^9^/L, they were 9% and 22%, respectively. After adjusting for injury severity, a higher platelet count was associated with decreased odds of hospital mortality (OR 0.998 per unit, 95% CI 0.996–0.999) and 12-month mortality (OR 0.998 per unit, 95% CI 0.997–0.999) in patients with moderate-to-severe TBI. Compared to patients with a normal platelet count, patients with thrombocytopenia not receiving platelet transfusion had an increased risk of 12-month mortality (OR 2.2, 95% CI 1.6–3.0), whereas patients with thrombocytopenia receiving platelet transfusion did not (OR 1.0, 95% CI 0.6–1.7).

**Conclusion:**

Early thrombocytopenia occurs in approximately one-tenth of patients with TBI treated in the ICU, and it is an independent risk factor for mortality in patients with moderate-to-severe TBI. Further research is necessary to determine whether this is modifiable by platelet transfusion.

**Supplementary Information:**

The online version contains supplementary material available at 10.1007/s00701-022-05277-9.

## Introduction

Early coagulopathy is a well-recognized predictor of poor outcomes in patients with traumatic brain injury (TBI) [9, 11, 13, 18, 28, 31, 35]. Patients with severe TBI and concomitant coagulopathy have very high mortality rates; up to 66% die [9, 18]. This is likely due to the increased risk of intracranial hematoma expansion and brain edema progression, which result in increased intracranial pressure (ICP) and decreased cerebral perfusion pressure [3, 13, 18, 28].

Thrombocytopenia plays an important role in trauma-induced coagulopathy. It is associated with an up to ninefold adjusted risk of death in TBI patients [28]. Although a clear association between a low platelet count and mortality in TBI patients is recognized, the definition of thrombocytopenia varies among studies, ranging from a platelet count of < 50 to a count of < 150 × 10^9^/L [11, 35]. Still, a threshold of < 100 × 10^9^/L seems to be the most frequently used definition of thrombocytopenia, and this is also the target level referred to in the European trauma guidelines for patients with TBI [29]. Early thrombocytopenia in TBI patients may be due to the TBI itself [18], but it is more commonly induced by preexisting comorbidities leading to a low platelet count (e.g., alcohol consumption and liver disease).

Most previous studies have used admission coagulation parameters to define coagulopathy [12, 27]. However, delayed thrombocytopenia can develop in patients presenting with a normal platelet count [8, 22]. The platelet-count decline is steeper in patients with progressive hemorrhage [10], more severe injuries, and older age [23], indicating that a normal platelet count at admission might not be enough to rule out the risk of coagulopathy.

We aimed to examine the prevalence of thrombocytopenia within the first 24 h in the intensive care unit (ICU) and to determine its association with hospital and 12-month mortality in patients with TBI treated in the ICU. We hypothesized that a lower platelet count is independently associated with a higher risk of mortality and that the association is strongest in patients with more severe TBI. Further, we examined the prevalence of platelet transfusion and conducted an explanatory analysis to identify an optimal platelet-transfusion threshold.

## Methods

### Study design and study population

We conducted a multicenter retrospective observational cohort study using the Finnish Intensive Care Consortium (FICC) database. The FICC has been described in detail [26]. We included adult patients with TBI who were treated in four Finnish tertiary intensive care units (ICUs) between 2003 and 2019.

In Finland, all specialized neurocritical care of TBI patients is centralized in five tertiary ICUs, and four of these ICUs, covering approximately two-thirds of the Finnish population, participate in the FICC. The database includes prospectively collected nationwide data from Finnish ICUs but excludes data from some specialized units (e.g., Helsinki University Hospital’s neurosurgical ICU).

For this study, we screened for eligibility all the primary admissions with a diagnosis of TBI (identified by the Acute Physiology and Chronic Health Evaluation (APACHE) III and ICD-10 diagnostic codes) in the database [25]. We excluded patients younger than 18 years, those of foreign origin (no data on outcomes available), those with nonemergency admission, and those with missing data for hospital mortality, 12-month mortality, the Simplified Acute Physiology Score (SAPS) II score, the Glasgow Coma Scale (GCS), or the Sequential Organ Failure Assessment (SOFA) coagulation score.

### Data extraction and variable definition

We extracted the following variables from the FICC database: age, gender, preadmission functional status, diagnosis, type and year of admission, APACHE II score and variables, Therapeutic Intervention Scoring System (TISS) 76 and 28 score and variables, SAPS II score and variables, comorbidities (using SAPS II or APACHE II comorbidity scores), SOFA score and variables (including bilirubin count), GCS score (defined as the worst measured score during the first ICU day in accordance with the APACHE II definition), and ICU and hospital length of stay. Significant comorbidity was defined by SAPS II and APACHE II comorbidity scores, and the comorbidities included metastatic cancer, hematologic malignancy, acquired immunodeficiency syndrome, heart failure class IV (according to the New York Heart Association Functional Classification), liver cirrhosis, chronic lung disease, and dialysis-dependent kidney failure. The use of ICP monitoring and mechanical ventilation was derived from TISS variables.

We derived the lowest platelet count during the first 24 h of ICU admission from the SOFA coagulation variable. We defined a platelet count < 100 × 10^9^/L as thrombocytopenia [29]. Data on platelet transfusion for the entirety of the ICU stay were retrieved from TISS variables and were available for patients admitted during 2003–2017 (starting in 2018, the centers gradually transitioned from TISS-76 to TISS-28 scoring, and data regarding platelet transfusion were not routinely collected after the transition).

TBI severity was categorized as mild, moderate, or severe based on the GCS score (GCS 13–15, 9–12, and 3–8, respectively). Preadmission functional status was determined by a modified version of the World Health Organization/Eastern Cooperative Oncology Group (WHO/ECOG) classification, used by the FICC, which categorizes patients into four groups. We dichotomized preadmission functional status into two groups: independent in the activities of daily life and dependent in the activities of daily life.

### Outcomes

We used 12-month all-cause mortality as the primary outcome measure and hospital mortality as the secondary outcome measure. These were extracted from the FICC database.

### Statistical analyses

For the statistical analyses, we used Stata (version 15, StataCorp, College Station, TX). Unless otherwise noted, all the categorical variables are presented as counts with percentages, and all the nonparametric continuous data are presented as medians with interquartile ranges (IQRs). We analyzed continuous variables for normality distribution using the Shapiro–Francia *W* test. None of the tested continuous variables were normally distributed; hence, we used the nonparametric Mann–Whitney *U* test to test for differences in distribution between two groups and the Kruskal–Wallis test to test for differences in distribution between more than two groups. We tested for differences in categorical variables between groups using a two-sided *χ*^2^ test.

To assess the association between platelet count and risk of death, we used a multivariable logistic regression. To account for case mix, we adjusted for age, gender, GCS score (continuous), admission type (operative vs. nonoperative), admission year, chronic comorbidity (according to the APACHE II and SAPS II criteria), modified SAPS II score (not including age, admission type, GCS score, or chronic comorbidity). Admission year was included as a variable because it may indirectly capture unmeasured changes in intensive care during the long study period (2003–2019) but also other parts of the TBI care pathway not otherwise measured. The performance of the adjustment was tested by assessing the model’s area under the receiver operating characteristic curve (AUC) and Hosmer–Lemeshow goodness of fit.

In a subgroup analysis, we separately analyzed patients with mild TBI (GCS 13–15) and moderate-to-severe TBI (GCS 3–12). In the first sensitivity analysis, we adjusted for platelet transfusion in patients admitted during 2003–2017. In the second sensitivity analysis, we examined the association between interaction term “thrombocytopenia” (< 100 × 10^9^/L) and “platelet transfusion” (during ICU admission) and 12-month mortality. In the third sensitivity analysis, we adjusted for bilirubin level (defined as the most abnormal bilirubin value measured during the first day of ICU stay [33]) to account for the potential confounding effect of preexisting alcohol use and liver disease. We used Spearman’s rank correlation test to assess the correlation between bilirubin level and platelet count. Further, we conducted an explanatory analysis to identify the platelet threshold for predicting 12-month and hospital mortality using Youden’s J index, adjusting for the abovementioned factors.

We visualized the underlying association between platelet count and risk of death by drawing a locally weighted scatterplot smoothing curve.

The results are presented as odds ratios (ORs) with 95% confidence intervals (CIs). *p* values under 0.05 were considered statistically significant. Because of the low number of missing data per variable, we performed complete case analyses.

We report the results of the study according to STROBE recommendations for observational studies [1].

## Results

### Baseline characteristics

**Fig. 1:**
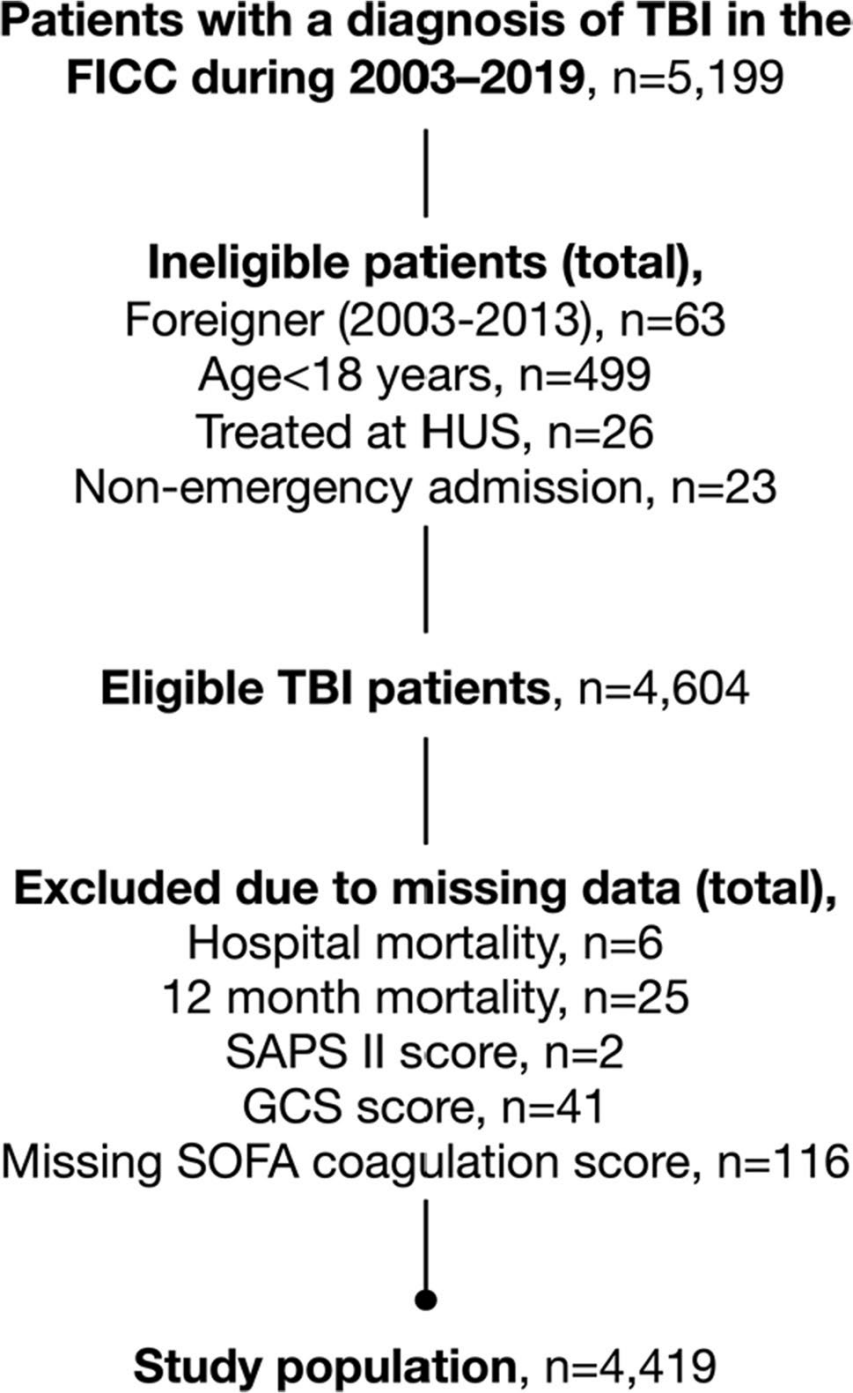
Flow chart of patient inclusion. Abbreviations: FICC Finnish Intensive Care Consortium, GCS Glasgow coma scale, HUS Helsinki University Hospital, SAPS Simplified Acute Physiology Score, SOFA Sequential Organ Failure Assessment, TBI traumatic brain injury

The study population consisted of 4419 patients (Fig. 1). The median age was 58 years, and 24% of the patients were female (Table 1). The overall prevalence of thrombocytopenia (platelet count < 100 × 10^9^/L) was 12% (*n* = 530). There was no change in platelet-count distribution during the study period (eFigure 1).

**Table 1:** Baseline characteristics for all patients and according to platelet count

Variable	All patients (*n* = 4419)	Platelet count (× 10^9^/L)	
		≥ 100 (*n* = 3889)	< 100 (*n* = 530)
Age, years	58 [44–69]	58 [44–69]	50 [50–67]
Female gender	1078 (24)	971 (25)	107 (20)
Functional dependency pre-admission^a^	464 (11)	411 (11)	53 (10)
Significant comorbidity	418 (10)	336 (9)	82 (15)
Operative admission	1370 (31)	1180 (30)	190 (36)
GCS score			
3–8	1942 (44)	1610 (41)	332 (63)
9–12	873 (20)	789 (20)	84 (16)
13–15	1604 (36)	1490 (38)	114 (22)
Platelet count, × 10^9^/L	181 [134–231]	192 (152–239)	80 (66–90)
Platelet transfusion during ICU stay	299 (7%)	118 (3%)	181 (34%)
SAPS II score	34 [23–49]	33 [22–47]	46 [32–60]
ICP monitoring	1013 (23)	879 (23)	134 (25)
Mechanical ventilation	2698 (61)	2285 (59)	413 (78)
ICU length-of-stay, days	1.7 [0.8–3.8]	1.7 [0.8–3.8]	1.8 [0.9–3.9]
Hospital length-of-stay, days	6 [3–10]	6 [3–10]	5 [2–9]
ICU mortality	308 (7)	216 (6)	92 (17)
Hospital mortality	501 (11)	361 (9)	140 (26)
12-month mortality	1109 (25)	854 (22)	255 (48)

Continuous variables are presented as median [IQR] and categorical variables as *n* (%)

*GCS* Glasgow coma scale, *ICP* intracranial pressure, *ICU* intensive care unit, *IQR* interquartile range, *N/A* not applicable, *SAPS* simplified acute physiology score

^a^Data missing for 137 patients

The overall hospital and 12-month mortality rates were 11% and 25%, respectively. In patients with thrombocytopenia, these rates were 26% and 48%, respectively (Table 1). In patients with a platelet count > 100 × 10^9^/L, the rates were 9% and 22%, respectively. In patients receiving a platelet transfusion during their ICU stay, the hospital and 12-month mortality rates were 16% and 42%, respectively. In patients not receiving a platelet transfusion, these rates were 11% and 24%, respectively. The predicted risk of hospital death decreased during the study period, indicating that the severity of TBI decreased with time (eFigure 2).

The comparison of baseline characteristics between 12-month survivors and nonsurvivors is shown in eTable 1, and the comparison between hospital survivors and nonsurvivors is shown in eTable 2. In general, survivors were younger, were more often independent in the activities of daily life, had fewer major comorbidities prior to admission, needed less mechanical ventilation, were less frequently monitored for ICP, had lower SAPS II scores, had higher GCS scores, and had higher median platelet counts than nonsurvivors.

Seven percent (*n* = 281/3882) of the patients received a platelet transfusion during their ICU stay (eTable 3). The median platelet count (documented during the first 24 h of ICU admission) for patients receiving a platelet transfusion was 88 × 10^9^/L (IQR 67–126). The intercenter range for the median platelet count for patients receiving platelet transfusion was 75–97 × 10^9^/L. All the patients with a platelet count of < 20 × 10^9^/L (5 out of 5 patients), 54% of the patients with a platelet count of 20–50 × 10^9^/L (32 out of 59 patients), and 48% of the patients with a platelet count of 50–100 × 10^9^/L (144 out of 322 patients) received a platelet transfusion during their ICU stay. In contrast, only 118 out of 3889 patients (3%) with a platelet count ≥ 100 × 10^9^/L received a platelet transfusion during their ICU stay. The frequency of platelet transfusion seemed to increase after 2010 (eTable 3).

### Multivariable analyses

The AUC for the severity-of-illness model for predicting 12-month mortality was 0.85 (95% CI 0.83–0.86), and the Hosmer–Lemeshow goodness-of-fit test *p* value was 0.315. The AUC for the severity-of-illness model for predicting hospital mortality was 0.91 (95% CI 0.90–0.92), and the Hosmer–Lemeshow goodness-of-fit test *p* value was 0.005. The Akaike information criterion (AIC) for 12-month-mortality prediction was 3563, and the AIC for hospital-mortality prediction was 1903. The Nagelkerke *R*^2^ values for 12-month-mortality prediction and hospital-mortality prediction were 0.29 and 0.40, respectively. The calibration belts showed good calibration for both 12-month- and hospital-mortality prediction (eFigure 3). Regarding hospital-mortality prediction, the model underpredicted for those with a risk of hospital death between 82 and 99% (more observed deaths than predicted deaths).

After adjusting for case mix, a higher platelet count was associated with decreased odds of 12-month and hospital mortality (Table 2). A subgroup analysis showed that the association between platelet count and mortality was the strongest in patients with a GCS of 3–12 (eTable 4). For patients with a GCS of 13–15, the platelet count was not associated with 12-month or hospital mortality (eTable 5).

**Table 2:** Results from the multivariable logistic regression analysis

Predictor	Adjusted OR (95% CI)	*p* value
	12-month mortality	
Age^a^	1.05 (1.04 to 1.06)	< 0.001
Female gender	0.86 (0.71 to 1.04)	0.124
GCS^a^	0.80 (0.79 to 0.82)	< 0.001
Significant comorbidity	2.21 (1.71 to 2.86)	< 0.001
Operative admission	0.82 (0.69 to 0.99)	0.036
Modified SAPS II score^a,b^	1.08 (1.07 to 1.10)	< 0.001
Admission year^a^	0.98 (0.96 to 1.00)	0.050
Platelet count, × 10^9^/L^a^	0.998 (0.997 to 0.999)	0.002
	Hospital mortality	
Age^a^	1.02 (1.01 to 1.03)	< 0.001
Female gender	0.88 (0.67 to 1.16)	0.370
GCS^a^	0.68 (0.64 to 0.71)	< 0.001
Significant comorbidity	1.57 (1.10 to 2.23)	0.012
Operative admission	0.65 (0.50 to 0.84)	0.001
Modified SAPS II score^a^^, b^	1.12 (1.10 to 1.14)	< 0.001
Admission year^a^	0.96 (0.93 to 0.98)	< 0.001
Platelet count, × 10^9^/L^a^	0.998 (0.996 to 0.999)	0.006

*CI* confidence interval, *GCS* Glasgow coma scale, *OR* odds ratio, *SAPS* simplified acute physiology score

^a^OR for one-unit increase in continuous variables

^b^SAPS II score excluding points for GCS, chronic disease, age, and admission type (operative vs non-operative)

**Fig. 2:**
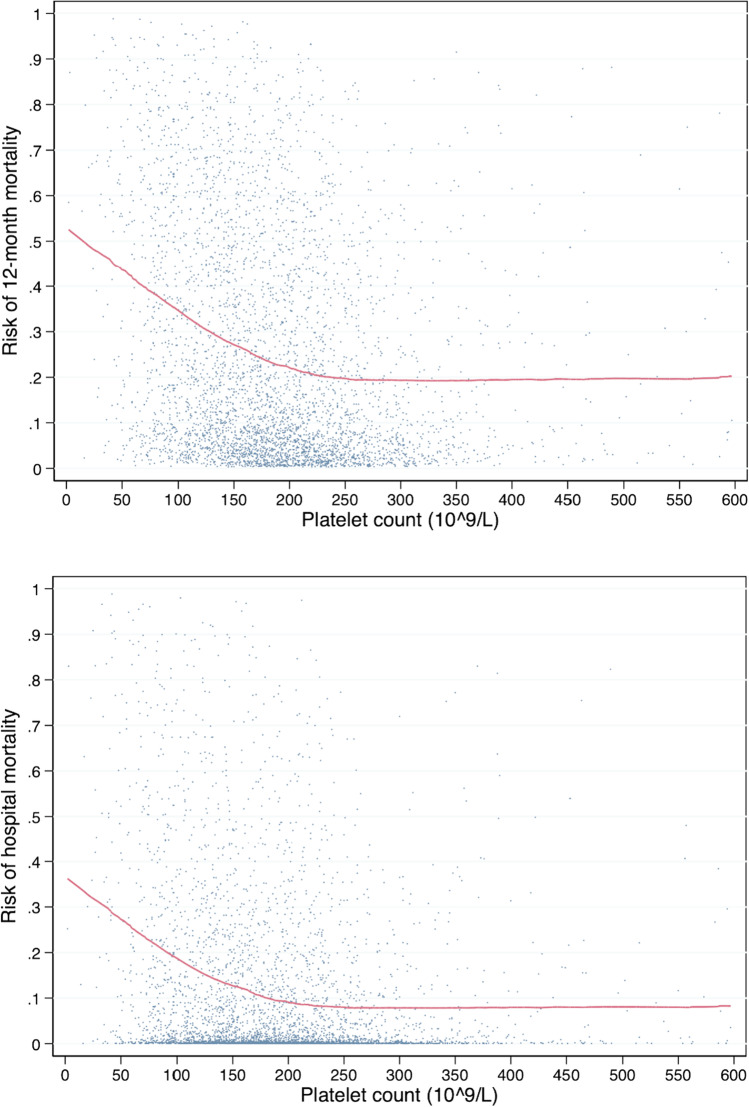
Locally weighted scatterplot smoothing (Lowess) curve visualizing the relationship between platelet count and predicted risk of 12-month (on top) and hospital (above) mortality in patients with GCS 3–12. A steep increase in the risk of death is seen with a platelet count below 200 × 10.^9^/L. Risk of death accounts for the variables in Table 2

The underlying relationships between platelet count and the predicted risk of 12-month and hospital mortality in patients with a GCS score of 3–12 are visualized in Fig. 2. An increase in the risk of 12-month and hospital mortality was observed when the platelet count was < 200 × 10^9^/L.

### Sensitivity analyses

In the first sensitivity analysis, adjusting for platelet transfusion in patients admitted during 2003–2017, the association between platelet count, hospital mortality, and 12-month mortality remained significant in the whole cohort (eTable 6) and in patients with GCS 3–12 (eTable 7). Platelet transfusion itself was associated with decreased odds of hospital death but not for 12-month mortality.

In the second sensitivity analysis, there was a significant interaction between early thrombocytopenia and platelet transfusion. Compared to patients with a normal platelet level, patients with thrombocytopenia not receiving a platelet transfusion had an increased risk of 12-month mortality (OR 2.2, 95% CI 1.6–3.0), whereas patients with thrombocytopenia receiving a platelet transfusion did not (OR 1.0, 95% CI 0.6–1.7) (Table 3). According to the Youden’s J index analysis, the platelet-level threshold for predicting 12-month mortality was 94 × 10^9^/L, and the threshold for predicting hospital mortality was 229 × 10^9^/L.

**Table 3:** Results from the multivariable logistic regression analysis including an interaction term

Variable	OR (95% CI)	*p* value
	12-month mortality	
Age^a^	1.05 (1.05–1.06)	< 0.001
Female	0.86 (0.71–1.04)	0.121
GCS^a^	0.81 (0.79–0.83)	< 0.01
Chronic comorbidity	2.14 (1.66–2.77)	< 0.001
Operative admission	0.82 (0.68–0.98)	0.032
Modified SAPS II^a, b^	1.08 (1.07–1.09)	< 0.001
Admission year^a^	0.98 (0.97–1.00)	0.106
Platelet count, × 10^9^/L^a^	1.00 (0.99–1.00)	0.783
Platelet transfusion	1.53 (0.99–2.38)	0.058
Platelet count, × 10^9^/L*Platelet transfusion interaction term		
≥ 100 * no transfusion	*Base*	
< 100 * no transfusion	2.18 (1.60–2.97)	< 0.001
< 100 * transfusion	0.97 (0.55–1.70)	0.912

No interaction term between platelet level ≥ 100 and platelet transfusion could be established due to the low number of patients receiving transfusion and strong correlation between no transfusion and normal platelet count

*CI* confidence interval, *GCS* Glasgow coma scale, *OR* odds ratio, *SAPS* simplified acute physiology score

^a^OR for one-unit increase in continuous variables

^b^SAPS II score excluding points for GCS, chronic disease, age, and admission type (operative vs non-operative)

In the third sensitivity analysis, there was a negative correlation between bilirubin and platelet count (*r* =  − 0.302, *p* < 0.001, eFigure 4). The multivariable logistic regression did not reveal a significant association between bilirubin level and mortality (eTable 8). The association between platelet count and 12-month mortality remained significant after adjusting for bilirubin level (OR 0.999, 95% CI 0.997–0.999, *p* < 0.001).

## Discussion

### Key results

In this large, multicenter retrospective study, we found that early thrombocytopenia occurred in 12% of the patients with TBI treated in the ICU. A higher platelet count during the first 24 h in the ICU was independently associated with decreased odds of hospital and 12-month mortality in patients with moderate-to-severe TBI (defined as a GCS score between 3 and 12). The association between thrombocytopenia and mortality was evident even for platelet levels under 200 × 10^9^/L. More specifically, our explanatory analysis suggested platelet-level thresholds of 94 × 10^9^/L and 229 × 10^9^/L for the association with an increased risk of 12-month mortality and hospital mortality, respectively. However, it remained uncertain whether early aggressive interventions (i.e., by platelet transfusion) aiming to avoid thrombocytopenia would improve outcomes, because early thrombocytopenia may be an indirect marker of higher TBI severity [6] or of generally more severe illness in patients with a poor overall prognosis.

### Comparison with previous studies and current guidelines

Our study’s findings regarding the overall prevalence of early thrombocytopenia and mortality rates are comparable to previous findings [4, 6, 11, 18, 27, 35]. The overall prevalence of coagulopathy, including other abnormalities in coagulation parameters, after TBI ranges from 7 to 63%, reflecting variations in the definition of coagulopathy but also the strong correlation between the prevalence of coagulopathy and brain-injury severity [18]. Coagulopathy occurs in more than 60% of patients with severe TBI, but it is uncommon in mild head injuries (< 1%) [18]. According to Schnüriger et al., in severe TBI patients, a platelet count < 100 × 10^9^/L was associated with a ninefold adjusted odds of death, and a platelet count < 175 × 10^9^/L was a significant predictor of hemorrhagic progression [28]. A CENTER-TBI analysis revealed a significant increase in risk of 6-month mortality among coagulopathic patients with isolated TBI (25%) compared to noncoagulopathic isolated-TBI patients (9%) [6]. In our study, hospital mortality was almost three times higher and 12-month mortality almost two times higher in thrombocytopenic TBI patients (26% and 48%, respectively) compared to nonthrombocytopenic patients (9% and 22%, respectively). This finding has at least two plausible explanations. First, a low platelet count might exacerbate the primary and secondary brain injury through hemorrhagic progression; second, a low platelet count could be an indirect marker of more severe primary brain injury. In support of the latter, we showed that thrombocytopenia was associated with mortality only in patients with moderate-to-severe TBI. Still, in this retrospective study, we could not confirm the association between a low platelet count and hemorrhagic progression and its effect on outcomes, because we did not have access to follow-up radiological images.

TBI patients can present with coagulopathy (such as thrombocytopenia) in the acute phase either due to preexisting disease (e.g., liver insufficiency, alcohol consumption), prior use of antithrombotic drugs, or trauma-induced coagulopathy itself. Although coagulopathy is a common finding in patients with TBI, it is crucial to point out that platelet count is just one parameter of the complexity of hemostasis. Hemostatic resuscitation in the early phases of treatment and prehospital pharmacotherapy might itself play a role in TBI-related coagulopathy. Tranexamic acid, the most commonly used antifibrinolytic agent, has not been clearly proven to improve outcomes in patients with TBI [2, 7]. A plausible explanation is that after TBI, both hypo- and hypercoagulability often occur sequentially or even simultaneously [18]. Further, a low platelet count does not necessarily indicate bleeding diathesis. For example, high fibrinogen levels have a strong concomitant effect on clot formation, and it has been suggested that impaired clot formation during thrombocytopenia might be partially compensated for by administrating fibrinogen concentrate [16, 32]. Moreover, it is likely that simultaneous coagulation-factor deficiency and thrombocytopenia, both of which play a major role in TBI-induced coagulopathy, have more devastating consequences than thrombocytopenia alone.

The cause of early thrombocytopenia in patients with TBI is multifactorial. On one hand, it has been proposed that TBI itself might cause thrombocytopenia and platelet dysfunction through pericontusional microthrombosis and platelet hyperactivity, which in turn lead to platelet consumption, depletion, and exhaustion [18]. These pathophysiological mechanisms can continue for several days after the initial insult. On the other hand, a significant number of patients suffering from TBI are under the influence of alcohol at the time of injury and/or have a history of chronic alcohol abuse. Up to 36–51% of patients treated for TBI are alcohol intoxicated at the time of injury, and up to 55–66% of TBI patients have a history of chronic alcohol abuse [24, 34]. Although bilirubin is not the most sensitive indicator of previous alcohol abuse, we demonstrated a clear, albeit moderate, association between early thrombocytopenia and bilirubin count. Because alcohol use and TBI are inextricably and bidirectionally linked and thrombocytopenia had developed in a very early phase of TBI (first 24 h of ICU admission), we believe previous alcohol abuse might be the most common etiology of thrombocytopenia in the present study cohort. Nevertheless, due to the retrospective nature of the study, no clear causal relationship could be defined, and we could not completely exclude the possibility of TBI-induced thrombocytopenia.

The role of platelet transfusion in TBI patients is challenging and controversial [20]. Although platelet transfusion is often considered for TBI patients with preinjury intake of antiplatelet therapy, the current data on its effects on platelet function and outcomes are inconclusive [18]. Even within Europe, there is substantial variation in the blood and coagulation management of patients with TBI treated in the ICU [15]. In our study cohort, 7% of patients received platelet transfusion, which is slightly lower in comparison to previous studies, where 12% of mild-to-severe TBI patients [4] and 35% of severe TBI patients were transfused with platelets [27]. The intercenter range for the median platelet count for patients receiving platelet transfusion was 75–97 × 10^9^/L in our study, but no clear causality could be inferred, because the median platelet count was derived from the first 24 h of admission, and the data on platelet transfusion included the entire ICU stay. European guidelines on major bleeding recommend maintaining a platelet count above 100 × 10^9^/L in patients with TBI, whereas the Brain Trauma Foundation guidelines provide no recommendations [29]. Still, not all patients with a platelet count < 100 × 10^9^/L received a platelet transfusion. This might be explained in part by possible treatment limitations made on some patients with a preexisting serious illness and/or a bad prognosis. Further, it is likely that thrombocytopenic TBI patients undergoing craniotomy tend to be transfused at lower thresholds than nonoperative patients. Interestingly, in our explanatory analysis, we found an optimal platelet threshold of 94 × 10^9^/L for predicting 12-month mortality, which is in line with the European guidelines, but the threshold for predicting hospital mortality was as high as 229 × 10^9^/L. It is possible that some patients with a normal platelet count were nonetheless transfused due to preinjury antiplatelet medication. However, platelet transfusion might have side effects, and it did not lead to improved outcomes in patients with spontaneous intracerebral hemorrhage using antiplatelet medication [5].

The efficacy of platelet transfusion has been evaluated in only a limited number of studies, most of which are retrospective and limited in sample sizes, patient stratifications, and confounding adjustments. Randomized controlled trials that examine platelet transfusions for different platelet-count thresholds are the only means of rigorously assessing the clinical efficacy of platelet transfusion in patients with TBI [30]. If outcomes are to be improved, future studies must identify strategies for optimizing coagulation profiles, including the optimal platelet-level thresholds. There is likely not a single definitive platelet threshold but rather a set of thresholds that depend on TBI type and underlying coagulation profile. This is highlighted by the relatively small effect size in this study. The small effect size for a one-unit increase in platelet count needs to be kept in mind while interpreting our results for clinical settings. Clearly, patients with a very low platelet count are at higher risk of increased mortality, but the overall risk of death is also determined by other risk factors and coagulation parameters.

### Strengths and limitations

We used a large multicenter database that collected prospective data from four university hospitals, covering approximately 70% of the Finnish population, during the 17-year period from 2003 to 2019. Thus, our results should be generalizable to similar settings. Our study’s low rate of missing data and outcomes strengthened its internal validity. We reported 12-month mortality in addition to hospital mortality, because the latter has been shown to severely underestimate mortality after TBI [21]. Further, by using the lowest platelet count within the first 24 h of ICU admission, we avoided the potential bias of timing of platelet testing, because patients may have a normal platelet count at admission but develop thrombocytopenia in the early phases of intensive care [8, 12, 17, 19, 22, 27]. The several sensitivity analyses we conducted showed similar results, strengthening the validity of our findings.

This study has some limitations. First, we were unable to obtain laboratory data regarding other coagulation parameters (e.g., prothrombin time, fibrinogen levels), platelet-function testing, or liver-function markers (e.g., aspartate amino transferase and alanine amino transferase). Data on prior antiplatelet-drug use and injury-severity scores were also unavailable. Moreover, the database lacked data on intracranial hemorrhage progression, a major factor leading to higher mortality rates in coagulopathy and thrombocytopenia. Second, our study was limited to mortality as the primary outcome instead of neurological outcome. Third, we were unable to control for the severity of extracranial injuries. It is possible that the association between platelet level and mortality differs between patients with an isolated TBI and those with additional major extracranial injuries [14].

## Conclusion

Early thrombocytopenia occurs in approximately one-tenth of patients with TBI treated in the ICU. A low platelet count within the first 24 h of ICU admission is associated with increased hospital and 12-month mortality. The association between thrombocytopenia and mortality is the strongest in patients with moderate-to-severe TBI. Prospective clinical trials are necessary to identify an individualized optimal platelet-transfusion threshold.

## Supplementary Information

Below is the link to the electronic supplementary material.

Supplementary file1 (DOCX 5.10 MB)

Supplementary file2 (DOCX 5.10 MB)

Supplementary file3 (DOCX 154 KB)

Supplementary file4 (DOCX 339 KB)

Supplementary file5 (DOCX 14.7 KB)

Supplementary file6 (DOCX 14.7 KB)

Supplementary file7 (DOCX 13.3 KB)

Supplementary file8 (DOCX 14.2 KB)

Supplementary file9 (DOCX 14.2 KB)

Supplementary file10 (DOCX 14.4 KB)

Supplementary file11 (DOCX 14.4 KB)

Supplementary file12 (DOCX 15.1 KB)

## Abbreviations

APACHE: Acute Physiology and Chronic Health Evaluation
AIC: Akaike information criterion
AUC: Area under the receiver operating characteristic curve
CI: Confidence interval
FICC: Finnish Intensive Care Consortium
GCS: Glasgow Coma Scale
ICD-10: International Classification of Diseases, Version 10
ICP: Intracranial pressure
ICU: Intensive care unit
IQR: Interquartile range
N/A: Non applicable
OR: Odds ratio
SAPS: Simplified Acute Physiology Score
SOFA: Sequential Organ Failure Assessment
TISS: Therapeutic Intervention Scoring System
TBI: Traumatic brain injury
WHO/ECOG: World Health Organization/Eastern Cooperative Oncology Group

## Declarations

The manuscript complies with instructions to authors. This manuscript has not been published elsewhere and is not under consideration by another journal. An abstract has been submitted to the 2022 Scandinavian Society of Anesthesiology and Intensive Care Medicine annual congress.

The authorship requirements have been met, and the final manuscript was approved by all authors.
