# Supplementary material for: Early thrombocytopenia is associated with an increased risk of mortality in patients with traumatic brain injury treated in the intensive care unit: a Finnish Intensive Care Consortium study
Source: Acta Neurochir (Wien). 2022 Jul 15;164(10):2731–40. doi: 10.1007/s00701-022-05277-9 (PMC9519714; doi:10.1007/s00701-022-05277-9)
Supplement: Supplementary file 1 — Supplementary file1 (DOCX 5.10 MB) [file 701_2022_5277_MOESM1_ESM.docx]

**eFigure 1.** Boxplot showing platelet count (x 10^9^/L) median with 25^th^ and 75^th^ percentiles throughout study period. No notable change in platelet count during the study period was noted.
