# Supplementary material for: Early thrombocytopenia is associated with an increased risk of mortality in patients with traumatic brain injury treated in the intensive care unit: a Finnish Intensive Care Consortium study
Source: Acta Neurochir (Wien). 2022 Jul 15;164(10):2731–40. doi: 10.1007/s00701-022-05277-9 (PMC9519714; doi:10.1007/s00701-022-05277-9)
Supplement: Supplementary file 2 — Supplementary file2 (DOCX 5.10 MB) [file 701_2022_5277_MOESM2_ESM.docx]

**eFigure 2**. The predicted risk of hospital mortality throughout study period, results are shown in medians with quartiles and adjacent values. The predicted risk of death decreased, indicating a decrease in the severity of TBI.
