# Supplementary figures and images for: Early thrombocytopenia is associated with an increased risk of mortality in patients with traumatic brain injury treated in the intensive care unit: a Finnish Intensive Care Consortium study
Source: Acta Neurochir (Wien). 2022 Jul 15;164(10):2731–40. doi: 10.1007/s00701-022-05277-9 (PMC9519714; doi:10.1007/s00701-022-05277-9)

**eFigure 3.** 12-month (upper) and hospital (lower) mortality calibration belt.

**
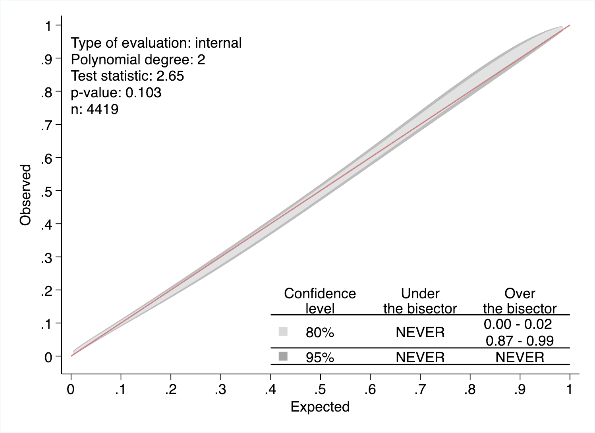
**

**
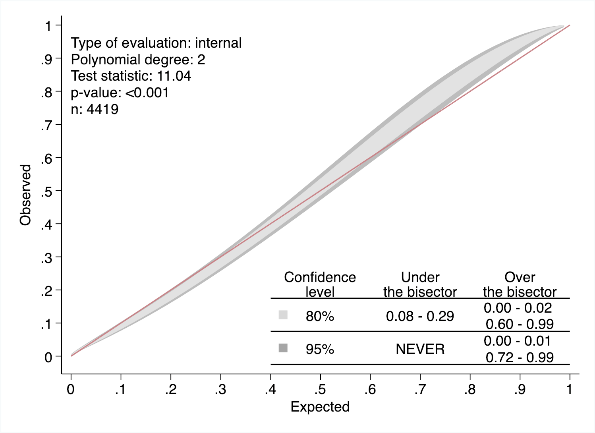
**

Supplement: Supplementary file 3 — Supplementary file3 (DOCX 154 KB) [file 701_2022_5277_MOESM3_ESM.docx]
