# Supplementary material for: Early thrombocytopenia is associated with an increased risk of mortality in patients with traumatic brain injury treated in the intensive care unit: a Finnish Intensive Care Consortium study
Source: Acta Neurochir (Wien). 2022 Jul 15;164(10):2731–40. doi: 10.1007/s00701-022-05277-9 (PMC9519714; doi:10.1007/s00701-022-05277-9)

**eFigure 4.** Scatter plot showing the correlation between bilirubin concentration and platelet count.


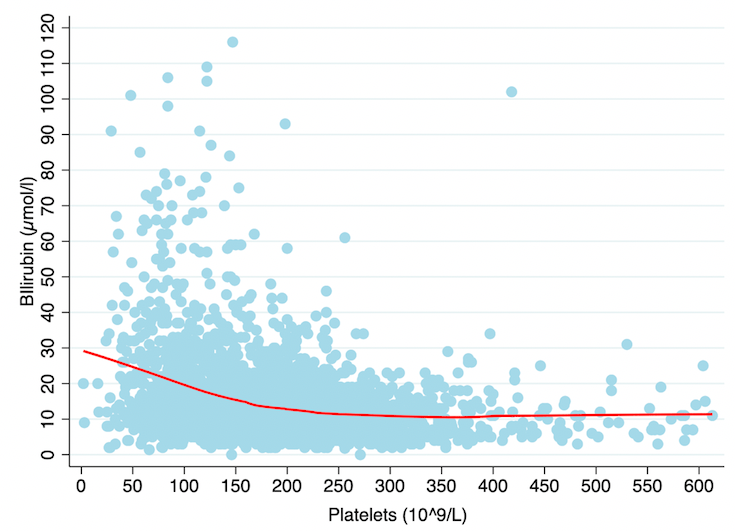

Supplement: Supplementary file 4 — Supplementary file4 (DOCX 339 KB) [file 701_2022_5277_MOESM4_ESM.docx]
