# Supplementary material for: Early thrombocytopenia is associated with an increased risk of mortality in patients with traumatic brain injury treated in the intensive care unit: a Finnish Intensive Care Consortium study
Source: Acta Neurochir (Wien). 2022 Jul 15;164(10):2731–40. doi: 10.1007/s00701-022-05277-9 (PMC9519714; doi:10.1007/s00701-022-05277-9)
Supplement: Supplementary file 5 — Supplementary file5 (DOCX 14.7 KB) [file 701_2022_5277_MOESM5_ESM.docx]

| **eTable 1:** Differences in patient characteristics between 12-month survivors and non-survivors | | | |
| --- | --- | --- | --- |
| **Variable** | **Survivors** | **Non-survivors** | ***p* value** |
| Number of patients (%) | 3310 (75) | 1109 (25) | N/A |
| Age, years | 56 [41-66] | 65 [54-75] | <0.001 |
| Female gender | 797 (24) | 281 (25) | 0.398 |
| Functionally dependent pre-admission^a^ | 272 (8) | 192 (18) | <0.001 |
| Significant comorbidity | 233 (7) | 185 (17) | <0.001 |
| Operative admission | 956 (29) | 414 (37) | <0.001 |
| Platelet count, x10^9^/L | 188 [145-237] | 154 [105-209] | <0.001 |
| Platelet count <100 x 10^9^/L | 275 (8) | 255 (23) | <0.001 |
| Platelet transfusion during ICU stay | 172 (5) | 127 (11) | <0.001 |
| GCS score |  |  |  |
| 3–8 | 1119 (34) | 823 (74) | <0.001 |
| 9–12 | 701 (21) | 171 (16) |  |
| 13–15 | 1490 (45) | 114 (10) |  |
| SAPS II score | 29 [20-41] | 54 [40-63] | <0.001 |
| ICP monitoring | 691 (21) | 322 (29) | <0.001 |
| Mechanical ventilation | 1754 (53) | 944 (85) | <0.001 |
| ICU length-of-stay, days | 1.7 [0.8-3.9] | 1.7 [0.9-3.7] | 0.432 |
| Hospital length-of-stay, days | 6 [4-11] | 4 [2-9] | <0.001 |
| Continuous variables are presented as median [IQR] and categorical variables as n (%)  Abbreviations: *GCS* Glasgow coma scale, *ICP* intracranial Pressure, *ICU* intensive care unit, *IQR* interquartile range, *N/A* not applicable, *SAPS* simplified acute physiology score  ^a^Data missing for 137 patients | | | |
