# Supplementary material for: Early thrombocytopenia is associated with an increased risk of mortality in patients with traumatic brain injury treated in the intensive care unit: a Finnish Intensive Care Consortium study
Source: Acta Neurochir (Wien). 2022 Jul 15;164(10):2731–40. doi: 10.1007/s00701-022-05277-9 (PMC9519714; doi:10.1007/s00701-022-05277-9)
Supplement: Supplementary file 6 — Supplementary file6 (DOCX 14.7 KB) [file 701_2022_5277_MOESM6_ESM.docx]

| **eTable 2**: Differences in patient characteristics between hospital survivors and non-survivors | | | |
| --- | --- | --- | --- |
| **Variable** | **Survivors** | **Non-survivors** | ***p* value** |
| Number of patients (%) | 3918 (89) | 501 (11) | N/A |
| Age, years | 58 [44-69] | 60 [50-70] | 0.002 |
| Female gender | 963 (25) | 115 (23) | 0.425 |
| Functionally dependent pre-admission^a^ | 408 (11) | 56 (12) | 0.581 |
| Significant comorbidity | 344 (9) | 74 (15) | <0.001 |
| Operative admission | 1210 (31) | 160 (32) | 0.631 |
| Platelet count, x10^9^/L | 185 [139-234] | 145 [94-198] | <0.001 |
| Platelet count <100 x 10^9^/L | 390 (10) | 140 (28) | <0.001 |
| Platelet transfusion during ICU stay | 251 (6) | 48 (10) | 0.008 |
| GCS score |  |  |  |
| 3–8 | 1478 (38) | 464 (93) | <0.001 |
| 9–12 | 849 (22) | 24 (5) |  |
| 13–15 | 1591 (40) | 13 (2) |  |
| SAPS II score | 31 [22-44] | 60 [52-67] | <0.001 |
| ICP monitoring | 846 (22) | 167 (33) | <0.001 |
| Mechanical ventilation | 2217 (57) | 481 (96) | <0.001 |
| ICU length-of-stay, days | 1.7 [0.9-4.0] | 1.2 [0.7-2.6] | <0.001 |
| Hospital length-of-stay, days | 6 [4-11] | 2 [1-5] | <0.001 |
| Continuous variables are presented as median [IQR] and categorical variables as n (%)  Abbreviations: *GCS* Glasgow coma scale, *ICP* intracranial pressure, *ICU* intensive care unit, *IQR* interquartile range, *N/A* not applicable, *SAPS* simplified acute physiology score  ^a^Data missing for 137 patients | | | |
