# Supplementary material for: Early thrombocytopenia is associated with an increased risk of mortality in patients with traumatic brain injury treated in the intensive care unit: a Finnish Intensive Care Consortium study
Source: Acta Neurochir (Wien). 2022 Jul 15;164(10):2731–40. doi: 10.1007/s00701-022-05277-9 (PMC9519714; doi:10.1007/s00701-022-05277-9)
Supplement: Supplementary file 7 — Supplementary file7 (DOCX 13.3 KB) [file 701_2022_5277_MOESM7_ESM.docx]

| **eTable 3**: Patients receiving platelet transfusion during ICU care | | | |
| --- | --- | --- | --- |
| **Variable** | **Patients receiving platelet transfusion / total number of patients, n (%)** | | |
| **Year** | **All patients**  **n=281** | **GCS score 3–12**  **n=229** | **GCS score 13–15**  **n=52** |
| 2003 | 6/145 (4%) | 6/121 (5%) | 0 |
| 2004 | 8/195 (4%) | 8/149 (5%) | 0 |
| 2005 | 3/200 (2%) | 3/161 (2%) | 0 |
| 2006 | 8/240 (3%) | 8/192 (4%) | 0 |
| 2007 | 11/245 (5%) | 10/187 (5%) | 1/58 (2%) |
| 2008 | 9/229 (4%) | 8/161 (5%) | 1/68 (2%) |
| 2009 | 14/238 (6%) | 13/177 (7%) | 1/61 (2%) |
| 2010 | 21/248 (9%) | 18/156 (12%) | 3/92 (3%) |
| 2011 | 30/328 (9%) | 29/209 (14%) | 1/119 (1%) |
| 2012 | 41/298 (14%) | 30/189 (16%) | 11/109 (10%) |
| 2013 | 28/286 (10%) | 20/158 (13%) | 8/128 (6%) |
| 2014 | 29/314 (9%) | 21/162 (13%) | 8/152 (5%) |
| 2015 | 26/316 (8%) | 18/181 (10%) | 8/135 (6%) |
| 2016 | 22/295 (8%) | 18/164 (11%) | 4/131 (3%) |
| 2017 | 25/305 (8%) | 19/166 (12%) | 6/139 (4%) |
| Platelet transfusion data available for 3,882 patients treated during 2003-2017. Values presented as patients receiving platelets/total number of patients in that specific group.  Abbreviations: *GCS* Glasgow coma scale, *ICU* intensive care unit | | | |
