# Supplementary material for: Early thrombocytopenia is associated with an increased risk of mortality in patients with traumatic brain injury treated in the intensive care unit: a Finnish Intensive Care Consortium study
Source: Acta Neurochir (Wien). 2022 Jul 15;164(10):2731–40. doi: 10.1007/s00701-022-05277-9 (PMC9519714; doi:10.1007/s00701-022-05277-9)
Supplement: Supplementary file 8 — Supplementary file8 (DOCX 14.2 KB) [file 701_2022_5277_MOESM8_ESM.docx]

| **eTable 4**: Results from the multivariable logistic regression analysis for patients with a GCS score of 3–12 | | |
| --- | --- | --- |
| **Variable** | **OR (95% CI)** | **p-value** |
|  | **12-month mortality** | |
| Age^a^ | 1.05 (1.04 to 1.06) | <0.001 |
| Female gender | 0.91 (0.73 to 1.12) | 0.367 |
| GCS^a^ | 0.80 (0.77 to 0.82) | <0.001 |
| Significant comorbidity | 2.02 (1.51 to 2.70) | <0.001 |
| Operative admission | 0.81 (0.67 to 0.99) | 0.040 |
| Modified SAPS II score^a,b^ | 1.09 (1.07 to 1.10) | <0.001 |
| Admission year^a^ | 0.98 (0.96 to 1.00) | 0.050 |
| Platelet count, x10^9^/L^a^ | 0.998 (0.997 to 0.999) | 0.004 |
|  | **Hospital mortality** | |
| Age^a^ | 1.02 (1.01 to 1.03) | <0.001 |
| Female gender | 0.87 (0.65 to 1.15) | 0.331 |
| GCS^a^ | 0.63 (0.59 to 0.67) | <0.001 |
| Significant comorbidity | 1.67 (1.16 to 2.40) | 0.006 |
| Operative admission | 0.64 (0.49 to 0.83) | 0.001 |
| Modified SAPS II score^a, b^ | 1.12 (1.10 to 1.14) | <0.001 |
| Admission year^a^ | 0.96 (0.93 to 0.98) | 0.015 |
| Platelet count, x10^9^/L^a^ | 0.998 (0.996 to 0.999) | 0.001 |
| Number of included patients, n=2,815  Abbreviations: *CI* confidence interval, *GCS* Glasgow coma scale, *OR* odds ratio, *SAPS* simplified acute physiology score  ^a^ OR for one-unit increase in continuous variables  ^b^ SAPS II score excluding points for GCS, chronic disease, age and admission type (operative vs non-operative) | | |
