# Supplementary material for: Early thrombocytopenia is associated with an increased risk of mortality in patients with traumatic brain injury treated in the intensive care unit: a Finnish Intensive Care Consortium study
Source: Acta Neurochir (Wien). 2022 Jul 15;164(10):2731–40. doi: 10.1007/s00701-022-05277-9 (PMC9519714; doi:10.1007/s00701-022-05277-9)
Supplement: Supplementary file 9 — Supplementary file9 (DOCX 14.2 KB) [file 701_2022_5277_MOESM9_ESM.docx]

| **eTable 5**: Results from the multivariable logistic regression analysis for patients with a GCS score of 13–15 | | |
| --- | --- | --- |
| **Variable** | **OR (95% CI)** | **p-value** |
|  | **12-month mortality** | |
| Age^a^ | 1.05 (1.04 to 1.07) | <0.001 |
| Female gender | 0.70 (0.43 to 1.15) | 0.161 |
| GCS^a^ | 0.65 (0.50 to 0.84) | 0.001 |
| Significant comorbidity | 3.08 (1.83 to 5.20) | <0.001 |
| Operative admission | 0.98 (0.59 to 1.61) | 0.928 |
| Modified SAPS II score^a,b^ | 1.04 (1.01 to 1.08) | 0.009 |
| Admission year^a^ | 0.98 (0.93 to 1.03) | 0.459 |
| Platelet count, x10^9^/L^a^ | 1.00 (1.00 to 1.00) | 0.273 |
|  | **Hospital mortality** | |
| Age^a^ | 1.04 (1.00 to 1.09) | 0.061 |
| Female gender | 1.11 (0.31 to 4.06) | 0.870 |
| GCS^a^ | 0.84 (0.39 to 1.80) | 0.656 |
| Significant comorbidity | 0.66 (0.08 to 5.82) | 0.712 |
| Operative admission | 0.90 (0.23 to 3.35) | 0.853 |
| Modified SAPS II score^a, b^ | 1.14 (1.07 to 1.22) | <0.001 |
| Admission year^a^ | 0.88 (0.76 to 1.01) | 0.068 |
| Platelet count, x10^9^/L^a^ | 0.99 (0.98 to 1.00) | 0.129 |
| Abbreviations: *CI* confidence interval, *GCS* Glasgow coma scale, *OR* odds ratio, *SAPS* simplified acute physiology score  ^a^ OR for one-unit increase in continuous variables  ^b^ SAPS II score excluding points for GCS, chronic disease, age and admission type (operative vs non-operative) | | |
