# Supplementary material for: Early thrombocytopenia is associated with an increased risk of mortality in patients with traumatic brain injury treated in the intensive care unit: a Finnish Intensive Care Consortium study
Source: Acta Neurochir (Wien). 2022 Jul 15;164(10):2731–40. doi: 10.1007/s00701-022-05277-9 (PMC9519714; doi:10.1007/s00701-022-05277-9)
Supplement: Supplementary file 10 — Supplementary file10 (DOCX 14.4 KB) [file 701_2022_5277_MOESM10_ESM.docx]

| **eTable 6**: Results from the multivariable logistic regression sensitivity analysis accounting for the effect of platelet transfusion on the association between platelet count and mortality | | |
| --- | --- | --- |
| **Variable** | **OR (95% CI)** | **p-value** |
|  | **12-month mortality** | |
| Age^a^ | 1.05 (1.04 to 1.06) | <0.001 |
| Female gender | 0.87 (0.71 to 1.07) | 0.198 |
| GCS^a^ | 0.80 (0.78 to 0.82) | <0.001 |
| Significant comorbidity | 2.10 (1.59 to 2.77) | <0.001 |
| Operative admission | 0.85 (0.71 to 1.03) | 0.105 |
| Modified SAPS II score^a,b^ | 1.08 (1.07 to 1.09) | <0.001 |
| Admission year^a^ | 0.97 (0.94 to 0.99) | 0.002 |
| Platelet transfusion | 1.32 (0.97 to 1.81) | 0.082 |
| Platelet count, x10^9^/L^a^ | 0.998 (0.998 to 0.999) | 0.014 |
|  | **Hospital mortality** | |
| Age^a^ | 1.02 (1.02 to 1.03) | <0.001 |
| Female gender | 0.89 (0.66 to 1.19) | 0.428 |
| GCS^a^ | 0.69 (0.65 to 0.72) | <0.001 |
| Significant comorbidity | 1.63 (1.13 to 2.37) | 0.009 |
| Operative admission | 0.65 (0.50 to 0.84) | 0.001 |
| Modified SAPS II score^a,b^ | 1.12 (1.10 to 1.14) | <0.001 |
| Admission year^a^ | 0.94 (0.91 to 0.97) | <0.001 |
| Platelet transfusion | 0.65 (0.43 to 1.00) | 0.049 |
| Platelet count, x10^9^/L^a^ | 0.997 (0.996 to 0.999) | 0.001 |
| Platelet transfusion data available for 3,882 patients treated during 2003-2017  Abbreviations: *CI* confidence interval, *GCS* Glasgow coma scale, *OR* odds ratio, *SAPS* simplified acute physiology score  ^a^ OR for one-unit increase in continuous variables  ^b^ SAPS II score excluding points for GCS, chronic disease, age and admission type (operative vs non-operative) | | |
