# Supplementary material for: Early thrombocytopenia is associated with an increased risk of mortality in patients with traumatic brain injury treated in the intensive care unit: a Finnish Intensive Care Consortium study
Source: Acta Neurochir (Wien). 2022 Jul 15;164(10):2731–40. doi: 10.1007/s00701-022-05277-9 (PMC9519714; doi:10.1007/s00701-022-05277-9)
Supplement: Supplementary file 11 — Supplementary file11 (DOCX 14.4 KB) [file 701_2022_5277_MOESM11_ESM.docx]

| **eTable 7**: Results from the multivariable logistic regression sensitivity analysis, in patents with a GCS of 3-12, accounting for the effect of platelet transfusion on the association between platelet count and mortality | | |
| --- | --- | --- |
| **Variable** | **OR (95% CI)** | **p-value** |
|  | **12-month mortality** | |
| Age^a^ | 1.05 (1.04 to 1.06) | <0.001 |
| Female gender | 0.89 (0.71 to 1.12) | 0.331 |
| GCS^a^ | 0.79 (0.76 to 0.82) | <0.001 |
| Significant comorbidity | 2.02 (1.49 to 2.75) | <0.001 |
| Operative admission | 0.86 (0.70 to 1.06) | 0.157 |
| Modified SAPS II score^a,b^ | 1.09 (1.07 to 1.10) | <0.001 |
| Admission year^a^ | 0.97 (0.94 to 0.99) | 0.004 |
| Platelet transfusion | 1.36(0.97 to 1.90) | 0.072 |
| Platelet count, x10^9^/L^a^ | 0.999 (0.997 to 0.999) | 0.013 |
|  | **Hospital mortality** | |
| Age^a^ | 1.02 (1.02 to 1.03) | <0.001 |
| Female gender | 0.87 (0.65 to 1.18) | 0.373 |
| GCS^a^ | 0.64 (0.60 to 0.68) | <0.001 |
| Significant comorbidity | 1.74 (1.19 to 2.56) | 0.004 |
| Operative admission | 0.64 (0.48 to 0.84) | 0.001 |
| Modified SAPS II score^a,b^ | 1.12 (1.10 to 1.14) | <0.001 |
| Admission year^a^ | 0.94 (0.91 to 0.97) | <0.001 |
| Platelet transfusion | 0.70 (0.45 to 1.08) | 0.103 |
| Platelet count, x10^9^/L^a^ | 0.998 (0.996 to 0.999) | 0.005 |
| Platelet transfusion data available for 2533 patients with a GCS of 3-12 treated during 2003-2017  Abbreviations: *CI* confidence interval, *GCS* Glasgow coma scale, *OR* odds ratio, *SAPS* simplified acute physiology score  ^a^ OR for one-unit increase in continuous variables  ^b^ SAPS II score excluding points for GCS, chronic disease, age and admission type (operative vs non-operative) | | |
