# Supplementary material for: Early thrombocytopenia is associated with an increased risk of mortality in patients with traumatic brain injury treated in the intensive care unit: a Finnish Intensive Care Consortium study
Source: Acta Neurochir (Wien). 2022 Jul 15;164(10):2731–40. doi: 10.1007/s00701-022-05277-9 (PMC9519714; doi:10.1007/s00701-022-05277-9)
Supplement: Supplementary file 12 — Supplementary file12 (DOCX 15.1 KB) [file 701_2022_5277_MOESM12_ESM.docx]

| **eTable 8:** Results from the multivariable logistic regression sensitivity analysis accounting for the effect of bilirubin on the association between platelet count and mortality | | |
| --- | --- | --- |
| **Predictor** | **Adjusted OR (95% CI)** | ***p* value** |
|  | **12-month mortality** | |
| Age^a^ | 1.05 (1.04 to 1.06) | <0.001 |
| Female gender | 0.86 (0.68 to 1.08) | 0.195 |
| GCS^a^ | 0.79 (0.77 to 0.81) | <0.001 |
| Significant comorbidity | 2.00 (1.47 to 2.71) | <0.001 |
| Operative admission | 0.81 (0.65 to 1.01) | 0.062 |
| Modified SAPS II score^a,b^ | 1.08 (1.06 to 1.10) | <0.001 |
| Admission year^a^ | 0.99 (0.97 to 1.01) | 0.049 |
| Platelet count, x10^9^/L^a^ | 0.999 (0.997 to 0.999) | 0.002 |
| Bilirubin count, μmol/L^a^ | 1.01 (1.00 to 1.01) | 0.054 |
|  | **Hospital mortality** | |
| Age^a^ | 1.02 (1.01 to 1.03) | <0.001 |
| Female gender | 0.96 (0.70 to 1.33) | 0.818 |
| GCS^a^ | 0.63 (0.59 to 0.68) | <0.001 |
| Significant comorbidity | 1.62 (1.07 to 2.45) | 0.023 |
| Operative admission | 0.68 (0.50 to 0.92) | 0.012 |
| Modified SAPS II score^a, b^ | 1.12 (1.10 to 1.14) | <0.001 |
| Admission year^a^ | 0.96 (0.94 to 0.99) | 0.016 |
| Platelet count, x10^9^/L^a^ | 0.999 (0.997 to 1.000) | 0.085 |
| Bilirubin count, μmol/L^a^ | 1.00 (0.99 to 1.00) | 0.614 |
| Abbreviations: *CI* confidence interval, *GCS* Glasgow coma scale, *OR* odds ratio, *SAPS* simplified acute physiology score  ^a^ OR for one-unit increase in continuous variables  ^b^ SAPS II score excluding points for GCS, chronic disease, age and admission type (operative vs non-operative) | | |
